# Comparison of Muscle MEPs From Transcranial Magnetic and Electrical Stimulation and Appearance of Reflexes in Horses

**DOI:** 10.3389/fnins.2020.570372

**Published:** 2020-09-25

**Authors:** Sanne Lotte Journée, Henricus Louis Journée, Hanneke Irene Berends, Steven Michael Reed, Cornelis Marinus de Bruijn, Cathérine John Ghislaine Delesalle

**Affiliations:** ^1^Equine Diagnostics, Wyns, Netherlands; ^2^Department of Virology, Parasitology and Immunology, Research Group of Comparative Physiology, Faculty of Veterinary Medicine, Ghent University, Merelbeke, Belgium; ^3^Department of Neurosurgery, University Medical Center Groningen, University of Groningen, Groningen, Netherlands; ^4^Department of Orthopedics, University Medical Center Utrecht, Utrecht University, Utrecht, Netherlands; ^5^Department of Orthopedics, University Medical Center Amsterdam, Amsterdam, Netherlands; ^6^Rood & Riddle Equine Hospital, Lexington, KY, United States; ^7^M.H. Gluck Equine Research Center, Department of Veterinary Science, University of Kentucky, Lexington KY, United States; ^8^Wolvega Equine Clinic, Oldeholtpade, Netherlands

**Keywords:** horses, transcranial stimulation, neurology, startle reflex, motor potentials, TES, TMS

## Abstract

**Introduction:**

Transcranial electrical (TES) and magnetic stimulation (TMS) are both used for assessment of the motor function of the spinal cord in horses. Muscular motor evoked potentials (mMEP) were compared intra-individually for both techniques in five healthy horses. mMEPs were measured twice at increasing stimulation intensity steps over the extensor carpi radialis (ECR), tibialis cranialis (TC), and caninus muscles. Significance was set at *p* < 0.05. To support the hypothesis that both techniques induce extracranially elicited mMEPs, literature was also reviewed.

**Results:**

Both techniques show the presence of late mMEPs below the transcranial threshold appearing as extracranially elicited startle responses. The occurrence of these late mMEPs is especially important for interpretation of TMS tracings when coil misalignment can have an additional influence. Mean transcranial motor latency times (MLT; synaptic delays included) and conduction velocities (CV) of the ECR and TC were significantly different between both techniques: respectively, 4.2 and 5.5 ms (MLT_*TMS*__–_-MLT_*TES*_), and −7.7 and −9.9 m/s (CV_*TMS*_-CV_*TES*_). TMS and TES show intensity-dependent latency decreases of, respectively, −2.6 (ECR) and −2.7 ms (TC)/30% magnetic intensity and −2.6 (ECR) and −3.2 (TC) ms/30V. When compared to TMS, TES shows the lowest coefficients of variation and highest reproducibility and accuracy for MLTs. This is ascribed to the fact that TES activates a lower number of cascaded interneurons, allows for multipulse stimulation, has an absence of coil repositioning errors, and has less sensitivity for varying degrees of background muscle tonus. Real axonal conduction times and conduction velocities are most closely approximated by TES.

**Conclusion:**

Both intracranial and extracranial mMEPs inevitably carry characteristics of brainstem reflexes. To avoid false interpretations, transcranial mMEPs can be identified by a stepwise latency shortening of 15–20 ms when exceeding the transcranial motor threshold at increasing stimulation intensities. A ring block around the vertex is advised to reduce interference by extracranial mMEPs. mMEPs reflect the functional integrity of the route along the brainstem nuclei, extrapyramidal motor tracts, propriospinal neurons, and motoneurons. The corticospinal tract appears subordinate in horses. TMS and TES are interchangeable for assessing the functional integrity of motor functions of the spinal cord. However, TES reveals significantly shorter MLTs, higher conduction velocities, and better reproducibility.

## Highlights

– Specifically in fright animals, such as horses, both TMS and TES can elicit SRs, visible as dominating middle and late mMEPs, which already are observed below transcranial motor thresholds.
– When, in both TMS and TES, mMEPs are examined without knowing that the stimulation intensity is below the threshold for TS, then mMEP latencies can be misinterpreted as being from intracranial origin and wrongfully labeled as pathologically prolonged.
– MLTs reflect the integrity of the spinal motor tracts that are essential for the control of motor function. These are in man and primates the corticospinal tract, however, in horses, they importantly co-act with extrapyramidal motor tracts up to the PN at the C3–C4 level that control muscle activity in the limb.
– TMS and TES are interchangeable techniques for measurement of transcranial motor latencies and conduction velocities in horses when one has to be aware of a bias of several milliseconds more in TMS, whereas TES offers a better reproducibility. Axonal conduction times and conduction velocities are most closely approximated by TES because intracortical synaptic delays can be excluded.
– When excluding delays other than net axonal conduction delays from the compound latency time, maximum axonal motor conduction velocities in the spinal cord of horses may exceed 100 m/s and forecast the presence of axonal diameters of at least 15 to 20 μm.
– mMEP amplitudes and waveforms have a limited clinical diagnostic meaning because the first part of pure transcranial mMEP waves only can be analyzed reliably within the TCW without interference by extracranially elicited mMEP components. A ring block is highly recommended in both TES and TMS to reduce the interference problem by extracranial mMEPs.

## Introduction

Transcranial stimulation (TS), either magnetic or electrical, has become a standard technique for assessment of the motor function of the human spinal cord by measuring either epidural or muscle motor evoked potentials (mMEPs) (MacDonald et al., 2013; Ziemann, 2017). Mayhew and Washbourne (1996) introduced this technique in the equine community using transcranial magnetic stimulation (TMS) and measuring mMEPs. Since then, TMS has evolved as a diagnostic tool in horses (Nollet et al., 2002, 2003a, 2004; Rijckaert et al., 2018). Recently, TES was introduced as a valuable alternative for TMS, to assess the functional integrity of the spinal cord in horses (Journée et al., 2018). TES and TMS share many neurophysiological properties, however, there are also important differences. These differences are mainly attributable to dissimilarities in physical–neural interfaces between both techniques. TMS applies brain stimulation by creating a magnetic field with a magnetic coil placed on the forehead of the horse. TES uses electrical currents through scalp electrodes to achieve brain stimulation (Boyd et al., 1986; Hess and Ludin, 1988; Deletis, 1993; Nollet et al., 2003d, 2005). Although differences can be expected, standardized studies comparing both techniques are currently lacking.

What is known at this point is that sedatives can reduce the success rate of both TES and TMS because of their hyperpolarizing effects, which may suppress the synaptic transmission to motoneurons (MNs; Nicoll and Madison, 1982; Zentner et al., 1992, 1997; Zhou et al., 1998; Sloan and Heyer, 2002). With TES, this can be compensated by the application of multipulse stimulation (Journée et al., 2007). Multipulse TES has also shown to be effective in horses (Journée et al., 2015).

**FIGURE 1:**
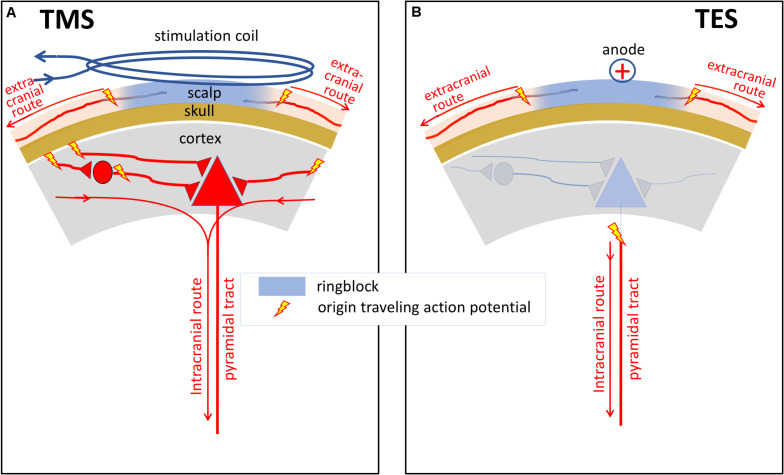
Schematic illustrations of differences between sites of activation and neural processing of neural elements under a TMS coil **(A)** and under anodal stimulation in TES **(B)** in human and primates. The major action of TES is to stimulate corticospinal tract axons directly, probably in the subcortical white matter. In contrast, the major action of TMS is the excitation of intracortical axons, which then cause indirect, transynaptic excitation of corticospinal and other corticofugal neurons. The curved lines with arrows show the intra- and extracranial routes of action potentials from the onset of axonal activation. Activation of the extracranial axons occurs outside the ring block.

**FIGURE 2:**
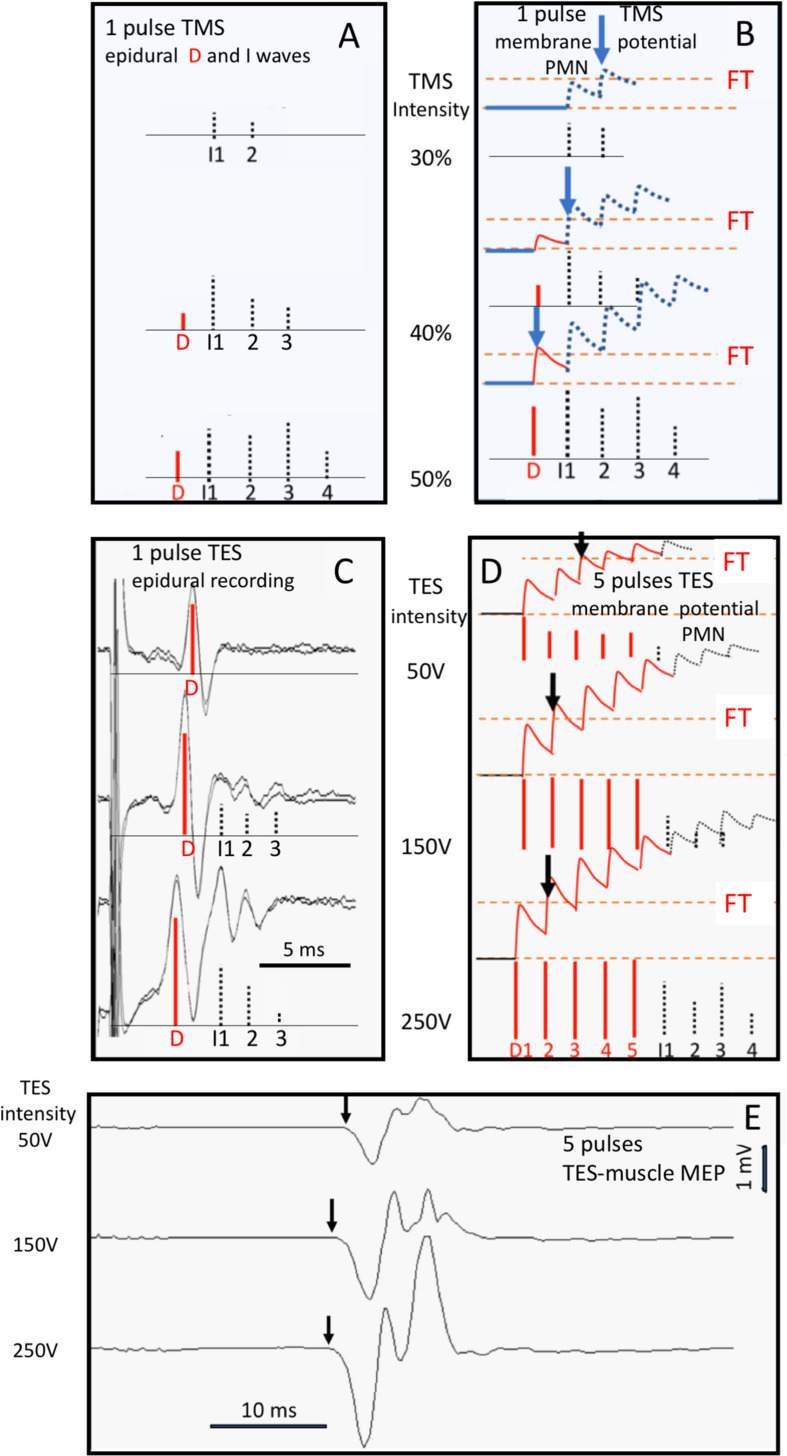
Human data visualizing the relation between D- and I-waves of TMS **(A,B)** and TES **(C–E)** mMEPs and the shortening effects on MLTs at three stimulation intensities: respectively, 30, 40, and 50% for TMS and 50, 150, and 250 V for TES. The vertical arrows in E point at the MLTs of the mMEPs. D-waves are indicated by red vertical bars and I-waves by gray bars. **(A)** Bars for which the height represents the size of epidural D- and I-waves from single-pulse TMS are reconstructed from the epidural recordings of Kaneko et al. (1996) **(C)** intra-operative epidural MEP at single-pulse TES and **(E)** mMEP response at five pulses per train; ipi = 1.3 ms. **(B,D)** are artist impressions of the course of EPSP summations of the MN membrane potentials depicted in graphs **(A,C)**. **(C–E)** Belong to a clinical patient (Department of Neurosurgery, UMCG, University of Groningen, Netherlands) under propofol/sufentanil anesthesia during intra-operative monitoring with **(C)** single-pulse TES epidural descending volleys and **(E)** multipulse TES mMEP recordings with five pulses per train of the same patient; ipi = 1.3 ms. The vertical arrows in panels **(B,D)** indicate the first crossings of the imaginary EPSP stair function (the abortion of the stair function by a transition into a firing action potential is not visualized for didactic reasons) at the FT.

Both TMS and TES generate trains of action potentials at the entry of descending motor tracts. From there, both techniques share the same pathways on the route to muscles from which mMEPs are recorded. However, in the brain, differences between sites of activation and neural processing of stimulation have been reported for TMS and TES in human and primates (Figures 1A,B; Boyd et al., 1986; Hess and Ludin, 1988; Deletis, 1993; Nollet et al., 2003d, 2005). The major action of TMS focuses on the brain cortex. TMS elicits excitation of intracortical axons, which then cause indirect, *trans*-synaptic repetitive excitation of corticospinal and other corticofugal neurons (Figure 1A). Cortical pyramidal neurons also deliver a train of action potentials to the corticospinal tract. These action potentials can be recorded epidurally as I (indirect) waves as shown in Figure 2A. These typically start with small amplitudes, which gradually increase with a linear increase of the stimulation intensity. At high intensities, a few corticospinal axons may become activated from which small epidural direct D (direct) waves arise as shown in Figure 2B. The membrane potentials of MNs show a stepwise increase modulated by the summation of excitatory potentials (EPSP) starting at each I- or, when present, D-wave. The increase of the stair function is proportional to the amplitude of the epidural waves, which, in turn, is proportional to the TMS intensity. This means that the membrane potential of an MN reaches its firing threshold (FT) at an earlier stage at higher intensities, which, in turn, implies a reduction of the muscular transcranial magnetic motor latency time (MLT) as shown in Figure 2B. The magnitude of the reduction encompasses one or two synaptic delays and is limited to about 3 ms. For example, the maximal intensity-dependent decrease of the MLT in dogs for TMS is reported to be at most 2.5 ms (Sylvestre et al., 1993).

In contrast to TMS, the major action of TES is to stimulate corticospinal tract axons directly in the subcortical white matter (Figure 2D). The generated action potentials can be recorded as a predominant D-wave as shown in Figure 2C. When the stimulation intensity increases, the D-waves reach their supramaximal amplitudes quickly although relative small I-waves may arise from additional activation of axons localized within the cortex itself. Multipulse TES introduces manifestation of additional D-waves as illustrated in Figure 2D. For example, at 150 V, the D-waves have reached their supramaximal amplitude. Between 50 and 150 V, there is a gradual increase of amplitude in which the FT arrival time shifts from the third back to the second D-wave. This is visible as a reduction of the transcranial electrical muscle MLT (EL) of about 1 ms from the first to the second mMEP (Figure 2E). Above a stimulation intensity of 150 V, the D-wave amplitudes are supramaximal and lock the FT arrival time at the second D-wave. Thereafter, the further reduction of the EL of about 0.3 ms at 250 V is ascribed to deeper stimulation in the brain. The latencies of D-waves decrease by 0.2 to 0.8 ms when the activation depth reaches the cerebral peduncle of human and primates, whereas I-wave latencies remain unaltered (Burke et al., 1990; Edgley et al., 1990; Rothwell et al., 1994; Nielsen et al., 1995; Li et al., 2007). D-waves can be activated as deep as the foramen magnum, where D-wave latencies are shortened by 1.8 ms (King, 1911; Hess and Ludin, 1988). Stimulation intensity-dependent MLT decreases have only been described in horses for multipulse TES and not for TMS (Journée et al., 2018).

Small differences of a few milliseconds of MLT between TMS and TES are observed in mMEPs, but mMEP amplitudes are about equal to each other (Boyd et al., 1986). Because conduction velocities depend on MLTs, these are also expected to differ between TMS and TES. In human and primates, MLTs of D-waves from TES are about 1.5–2 ms shorter when compared to TMS, and D-wave amplitudes are larger. The latencies of the dominating D-waves in TES are about 1.5 ms shorter than the first I-wave, which is the size of one synaptic delay (Boyd et al., 1986; Hess and Ludin, 1988; Edgley et al., 1997). The MLT is longer than EL because, in TMS, I-waves have a dominating role.

When motor conduction velocities are obtained from division of MLTs by the traversed distance from stimulation to recording electrodes (Mayhew and Washbourne, 1996), conduction velocities are also different between both techniques. These are a compound result of the conduction velocities of spinal motor tracts and peripheral nerves and depend on the sum of synaptic delays of an unknown number of interposed neurons and a neuromuscular junction. The specific intraspinal conduction velocities of motor tracts can be estimated when the number of interneurons and the length of the central and peripheral routes are known.

Finally, when comparing mMEP parameters for TMS and TES in previously published equine studies, it is important to realize that MLTs and also mMEP amplitudes depend on many additional factors, such as sedation and level of MN facilitation. MLTs have been reported to be dependent on height at withers, temperature, sedation, and MN modulation by muscular activity (Amassian et al., 1992; Houlden et al., 1999; Sloan and Heyer, 2002; Nollet et al., 2003b; Lemon and Griffiths, 2005).

Until now, no standardized study has been available comparing TMS and TES output. Many of the aforementioned variables can be standardized across both techniques when mMEPs evoked by TMS and TES are compared within the same horse.

Interestingly, specifically in horses, TES studies reveal expression of late mMEP responses in all extremities, probably due to reflexes resembling startle responses (SR) caused by TES acting extracranially on somatosensory afferents as shown in Figure 1B. These late mMEP responses are already visible below the transcranial motor threshold intensities as shown by Journée et al. (2015). Above these thresholds, these are prominently present and they strongly interfere with transcranial mMEPs when assessment is performed beyond the transcranial time window. Because TMS also activates extracranial sensory axons (see Figure 1A), it is hypothesized that TMS elicits similar late mMEPs. Their inevitable prominent presence gives reason for further exploration by reviewing the literature.

The goals of the current study are (1) to verify the hypothesis that late mMEPs are elicitable in horses by both TMS and TES below transcranial stimulation thresholds. They most likely result from extracranially evoked brain stem reflexes appearing as SRs. By reviewing the literature, occurrence of brain stem and high cervical reflexes are explored in TMS in relation to late mMEPs. (2) We aim to check whether normative data for mMEP latencies, motor conduction velocities (MCV), and MEP amplitudes from both techniques can be considered interchangeable; (3) to check whether significant left-to-right body side differences can be found for these mMEP parameters within and between both techniques; and (4) to compare reproducibility of measurements between TES and TMS techniques.

## Materials and Methods

### Materials

The study protocol was approved by the animal ethics committee of the University of Groningen, the Netherlands, and registered as DEC6440B.

The study group consisted of five horses (three geldings, two mares) aged between 4.2 and 20.5 years (mean: 11.1 ± 7.1 years) with a height at withers of 160.0 ± 7.0 cm and weight 542 ± 74 kg.

### Methods of Measurement

All measurements were performed in the Wolvega Equine Clinic in Oldeholtpade, The Netherlands. Horses were prepared as previously described (Journée et al., 2015). Sedation was performed in all horses, each time by i.v. administration of detomidin (Detosedan)^1^ and butorphanol (Butomidor)^2^ (both 1.5–2.0 mcg/kg in total).

**FIGURE 3:**
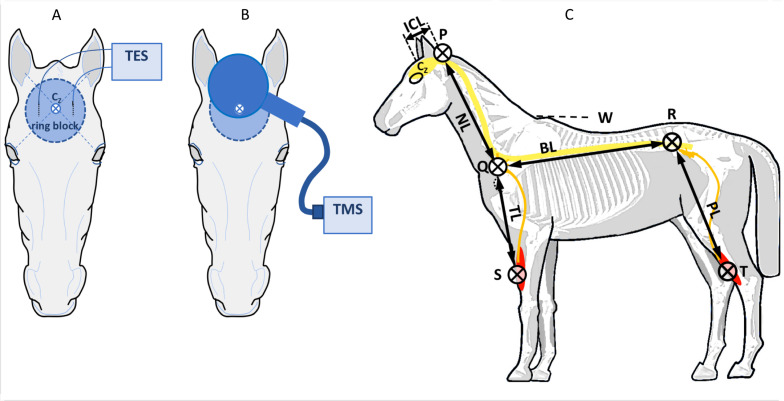
Placement of TES needle electrodes **(A)** and TMS coil **(B)** on the head of a horse. The vertex is defined at the cross-section of connecting lines between the ears and contralateral eyes. Circular area: ring block. **(C)** Anatomic landmarks for estimation of the axonal lengths in the spinal cord and peripheral nerves: P, occipital protuberance; Q, anterior rim of the scapula near corpus C7; R, dorso-ventral point of the hip; S, upper electrode ECR; T, upper electrode TC. ICL, intracranial segment length; NL, neck length 7 cervical corpora; BL, back length between Q and R; TL, length peripheral nerve limb; PL, length peripheral nerve hind limb.

A subcutaneous ring block surrounding Cz of about ∅ 8 cm was placed as shown in Figures 3A,B, using 300–400 mg lidocaine 2% + adrenaline.

For TES, two needle electrodes (L 35 mm, ∅0.45 mm, type RMN35/0.45 Electrocap BV, Nieuwkoop, Netherlands) were placed subcutaneously in a frontal direction 2.5 cm bilateral from the vertex at Cz as depicted in Figure 3A. TES was performed using biphasic multipulse trains of three pulses (constant voltage interpulse interval, ipi, = 1.3 ms), using a human intraoperative neurophysiological monitoring system (Neuro-Guard JS Center, Bedum, Netherlands).

Application of a voltage series consisting of 10-V steps starting at 0 V was selected out of the original voltage scheme presented in a previous study (Journée et al., 2018). TES was performed twice at each voltage. After reaching the transcranial electrical motor thresholds (ET), the stimulation was continued to ET + 50V. The transcranial stimulation threshold was defined at stimulation intensity (V for TES and percentage of maximum output for TMS) at the first occurrence of the early mMEP after the latency jump from the late to the early MEP.

Transcranial magnetic stimulation was applied through a circular coil (MC 125, Medtronic Functional Diagnosis A/S, Skovlunde, Denmark, maximum magnetic gradient 41 kT/s) placed symmetrically over the midline over the head with the lower rim about 2 cm frontal from Cz and connected to a MagPro Compact magnetic stimulator (Medtronic Functional Diagnosis A/S) as depicted in Figure 3B. Biphasic pulses of 0.28 ms were applied using a 10% stepwise increasing protocol, starting at 0%. TMS was performed twice at each step. When TMS motor thresholds (MT) were reached, stimulation was continued to MT + 50%.

Muscle motor evoked potentials were recorded bilaterally (left vs. right) from subcutaneous needle electrodes 82015-PT L 12 mm 27GA Rochester Lutz, FL, United States, in the forelimb in the musculus extensor carpi radialis (ECR) (10 and 20 cm above the os carpi accessorium), in the hind limb in the musculus tibialis cranialis (TC) (10 and 20 cm above the medial malleolus) and in the musculus caninus (CAN) (2 cm interspace). A ground needle electrode was placed subcutaneously in the neck. The signals were processed by highpass and lowpass filters of 50 and 2500 Hz (3 dB cutoff level) and digitally stored for later retrieval. The total length of the motor conduction route to the fore and hind limbs is estimated from segment lengths over the brain, spinal cord, and peripheral nerves as depicted in Figure 3C. Segment lengths were measured with a tape measure between anatomic landmarks for estimation of the axonal lengths for different sections: in the brain (intracranial segment: ICL), spinal cord (NL: neck, and BL: body segments), and peripheral nerves (thoracic: TL and pelvic: PL segments). The landmarks are described in the legend of Figure 3.

### Data Processing

For validation of the hypothesis that late mMEP components are extracranially elicited in both TMS and TES and how they relate to transcranial mMEPs as a function of transcranial stimulation intensities, landscape plots comparing pairs for TMS and TES of ECR and TC mMEPs are used. The somatotopic wideness of SRs of late mMEPs between brain stem and low lumbar segmental levels representing the SRs are shown by landscape plots of, respectively, CAN, ECR, and TC mMEPs for small TES intensity steps.

Considered mMEP parameters are motor latencies (MLTs), conduction velocities, and amplitudes of the ECR and TC for both TMS and TES. These were subjected to left versus right comparisons and a regression analysis to reveal the dependence of MLTs on stimulation intensities. The motor latencies are defined as the time lag between the onsets of the stimulation artifact of the TMS pulse or TES pulse train and mMEPs when these were unambiguously distinguishable from baseline noise from time readings using a cursor. The electrical conduction velocities (ECV) and MCVs are compound velocities that include central (CMCV) and peripheral axonal conduction velocities. ECV and MCV are derived by division of the traveled route lengths being equal to (ICL + BL + TL) for the thoracic and (ICL + NL + BL + PL) for the pelvic routes by the respective thoracic and pelvic mMEP latencies (Figure 3). The net axonal conduction times and velocities are estimated by correction for interneuron and neuromuscular synaptic delays. Intradural fractions of the thoracic and pelvic route lengths are computed as, respectively, (ICL + NL)/(ICL + NL + TL) and (ICL + NL + BL)/(ICL + NL + BL).

The mMEP amplitudes are defined as the maximum amplitude differences (top–top values) in the transcranial time window (TCW) as defined by the time region before the onset of the subthreshold late mMEP just before the stepwise transition to the transcranial mMEP.

### Statistical Analysis

Statistical analysis was performed with SPSS^TM^ software, version 20.0.0, IBM^TM^. The sequence of the TES and TMS measurement series was alternated between subsequent cases to minimize time-dependent bias effects in comparisons. Means are compared by paired *t*-tests, and a significance level of *p* ≤ 5% is applied throughout the study.

#### Comparison of mMEP Latencies and Compound Conduction Velocities Between TES and TMS

For each case *n* and muscle group *m*, mean electrical EL_*m,n*_ and magnetic ML_*m,n*,_ and standard deviations were computed over six data pairs of ECR and TC mMEPs from stimulation intensities at 10, 20, and 30 V above ET and 10, 20, and 30% above MT. The mean electrical and MLTs per muscle group and case, EL_*m*_ and ML_*m*_, with standard deviations were computed over all five cases. Mean electric and magnetic paired differences: mDL_*m*_ were computed from 30 recordings (five cases; six values/case).

#### Effect of Stimulation Intensity on mMEP Latencies for TES and TMS

The stimulation intensity dependence of EL_*m*_s and ML_*m*_s are estimated by linear regression analysis between ET + 10 and 40 V and between MT + 10% and 40%. The number of points in each scatterplot is 40 (five cases; eight points/case). For the computation of the slope of the regression line and correlation, the mean MT_*m,n*_ of the stimulation intensities are subtracted from MT_*m,n*,_*_*i*_* (*i* is the stimulation intensity variable) prior to the computations. This excludes the influence of interindividual ML and EL variations, which is in favor of a minimal scatter variance and optimal correlation and significance.

#### Comparison of mMEP Amplitudes for TES Versus TMS

Mean mMEP amplitudes were compared for TMS versus TES for two muscle groups by a paired *t*-test. The mean amplitudes are computed over the five individual means per case, which were obtained between electrical and magnetic stimulation intensities of ET + 10 V and ET + 40 V and, respectively, MT + 10% and MT + 40%.

#### Left-to-Right Body Side Differences for mMEP Parameters for TES Versus TMS

Left–right differences for latencies and amplitudes were tested in mean values of five cases by a paired *t*-test.

#### Comparison of the Accuracy and Reproducibility of mMEP Latencies for TES Versus TMS

The accuracy ACL_*e*_ of the latency times of TES mMEPs is computed as the root of the mean squares (RMS) of the differences of latency pairs and their shared mean per stimulation intensity step, being the overall reproducibility RP_*e*_ divided by the mean of the mean latencies per step and case of (*EL1*(*case*,i) + *EL2*(*case*,i))/2 according to:

$${\text{ACL}}_{\text{e}}=$$

$$\frac{\sqrt{\sum_{\mathrm{case1}}^{\text{s}}\sum_{i=10\,V,\,s\,t\,e\,p\,10\,V}^{40\,V}{\left(\mathrm{EL1}\,\left(\mathrm{case},i\right)-\mathrm{EL2}\,\left(\mathrm{case},i\right)\right)}^{2}/40}}{\sum_{\mathrm{case1}}^{\text{s}}\sum_{i=10\,V,\,s\,t\,e\,p\,10\,V}^{40\,V}\left(\mathrm{EL1}\,\left(\mathrm{case},i\right)+\mathrm{EL2}\,\left(\mathrm{case},i\right)\right)/40}$$

×100%

In the nominator, the total number of EL values results from two squared values/step, four steps/case, and five cases. RP_*e*_ is the RMS deviation from the expectance value of a zero difference of EL latency-pairs. RP_*e*_ and ACL_*e*_ are insensitive to influences of stimulation intensities on latencies and differences between cases. The normalization to mean latencies by the denominator makes the reproducibility of ACL comparable with coefficients of variation. For the reproducibility and accuracy for TMS, a similar computation of the stimulus-to-stimulus reproducibility RP_*m*_ and accuracy ACL_*m*_ for magnetic latencies is achieved by replacing the units [V] by [%] and EL by ML.

## Results

### Presence of Extracranial Reflexes at Increasing Stimulation Intensities of TMS and TES

**FIGURE 4:**
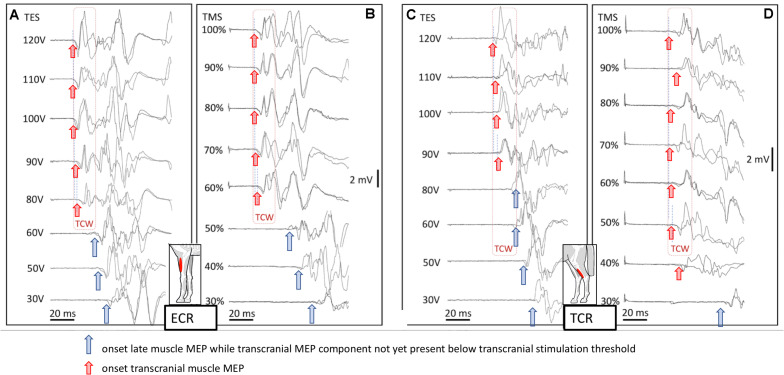
Landscape plots of muscle mMEPs of the m. ECR **(A,B)** and m. TC **(C,D)** at TES **(A,C)** and TMS **(B,D)**. TS thresholds: ET = 80 V (ECR) and 90 V (TC); MT = 60% (ECR) and 50% (TC). The TCW are indicated by the dashed box contour lines. The up-pointing arrows indicate the onset of transcranial or extracranial late mMEPs as described by the legend in the figure.

Figure 4 shows four mMEP landscape plots as a function of transcranial stimulation intensities for TES (A, C) and TMS (B, D) in the ECR and TC muscles. When following the graphs from bottom to top, late mMEPs become visible below the transcranial thresholds. As the stimulation intensity increases, the latency decreased markedly by about 10–40 ms. At transcranial threshold intensities, jump-wise decreases of 15–20 ms of ML and EL were noticeable, after which MLTs decreased by only a few ms. The latency jumps, which are typical for TES in horses (Journée et al., 2015, 2018), were also present after TMS. A TCW isolates the transcranial part of the mMEP from extracranial mMEP components.

**FIGURE 5:**
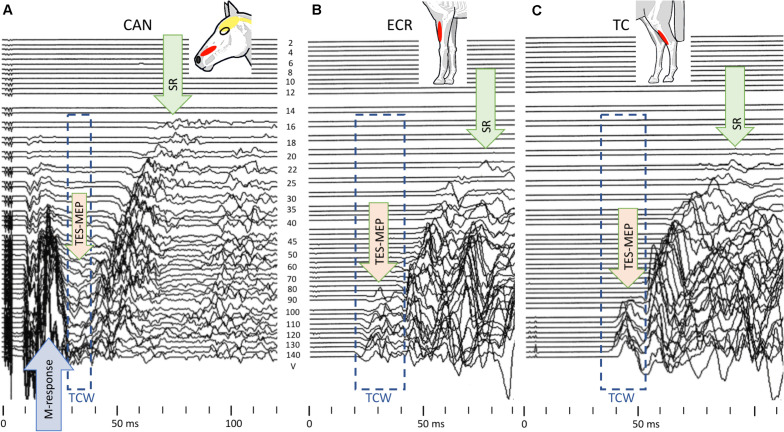
TES-mMEP landscape plots of the TES-mMEPs of the CAN **(A)**, ECR **(B)**, and TC **(C)** muscle groups illustrating the close relationship between stimulation threshold voltages of caninus muscle (CAN) responses (M-response) from extracranial elicited facial nerve axons **(A)** and the also assumed extracranial elicited SRs (example from case 4). Stimulation thresholds: M-response: 16 V, SR: for CAN 14 V; ECR and TC 22 V, and TES-mMEP all muscle groups: 80–90 V. Note that the TCW of the CAN mMEPs is smaller than for the two mMEP series due to squeezing by leading M-responses elicited by direct activation of facial nerve axons.

Figure 5 shows TES-mMEP landscape plots of the TES-mMEPs of the CAN (A), ECR (B), and TC (C) muscle groups to illustrate the relation between threshold voltages and responses resulting from extracranial excitation of motor axons in the facial nerve (A: M-response) and SR assumed to result from extracranial excitation of sensory afferents.

### MLTs

#### Comparison of MLTs TES Versus TMS

Latency times were significantly shorter and coefficient of variation was significantly smaller for TES when compared to TMS (Table 1). The overall mean latency times for TMS and TES are according to Table 1 for the ECR, respectively, 24.32 ± 1.23 (mean ± SD) and 20.14 ± 0.84 ms, and for the TC, respectively, 42.63 ± 3.48 and 37.32 ± 1.89 ms. All standard deviations are markedly higher for TMS when compared to TES. This difference is also reflected in the coefficients of variation for ML (TMS) and EL (TES), which are for the ECR and TC for, respectively, TMS: CV_*ML,ECR*_ = 5.5%, CV_*ML,TC*_ = 8.1%; and for TES: CV_*EL,ECR*_ = 4.2% and CV_*EL,TC*_ = 5.1%. All mean latencies of TMS are highly significantly greater than for TES. Their differences vary for the ECR between 4.22 and 4.34 ms and for the TC between 5.17 and 5.45 ms.

**TABLE 1:** Survey of mean and standard deviations (sd) of motor latency values ML, EL, mean ML-EL differences with significance sig for left (L), right (R), and intermediate values of left and right (L + R) of ECR and TC muscles (*n* = 5).

**Muscle group**		**TMS ML**					**TMS-TES difference ML-EL**			**TES EL**				
		**mean ms**	**sd ms**	**m ms/%**	**R^2^**	**sig**	**mean ms**	**sd ms**	**sig**	**mean ms**	**sd ms**	**m ms/V**	**R^2^**	**sig**
ECR	L	24.57	1.47				4.34	0.93	0.000	20.23	0.94			
	R	24.17	1.28				4.22	1.25	0.002	19.76	0.83			
	L and R	24.32	1.33	−0.085	0.704	0.000	4.27	1.08	0.001	20.14	0.84	−0.090	0.716	0.000
TC	L	42.28	3.13				5.17	1.41	0.001	37.11	1.78			
	R	42.98	3.85				5.45	2.40	0.007	37.53	2.11			
	L and R	42.63	3.48	−0.089	0.368	0.000	5.31	1.80	0.003	37.32	1.89	−0.107	0.647	0.000

*Furthermore, the slope m (units: ms/% for TMS; ms/V for TES), correlation R, and significance are given for the regression lines of ML and EL for 40 latency/TS intensity pairs in Figure 4 after correction of the ML or EL bias. All ML-ML differences and correlation factors are significant for *p* < 0.05.*

#### Motor Latency Differences Left Versus Right

Left-to-right differences of mean TMS and TES latency times ML and EL and standard deviation are for the ECR: ML = 0.40 ± 1.38 ms and EL = 0.47 ± 0.89 ms and for the TC: ML = −0.7 ± 3.51 and EL = −0.42 ± 1.95. All *p* values are >0.25, which implies, for TMS as well as for TES, no significant differences between the left and right sides of mMEP latency times in all muscle groups.

#### Dependence of Transcranial Latency Times on Stimulation Intensity

**FIGURE 6:**
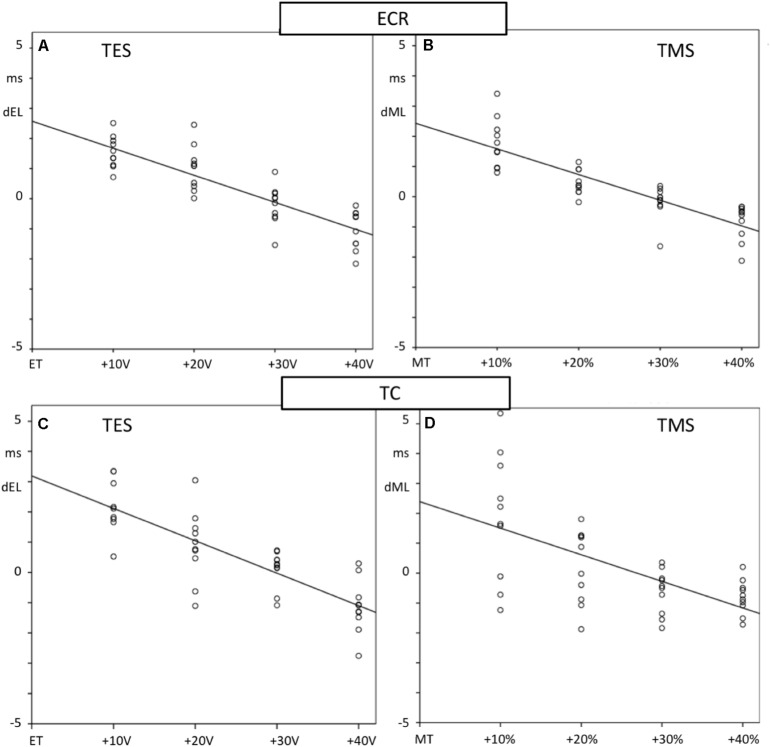
Scatter plots showing the regression lines of MLTs of fore limb **(A,B)** and hind limb **(C,D)** mMEPs and TS intensities of TES **(A,C)** and TMS **(B,D)**. The TES increases are given as voltage differences with the stimulation thresholds of ET for electrical and as percentage differences with MT for magnetic stimulation. Differences with mean MLTs, dEL for TES and dML for TMS, are plotted vertically and TS-intensities horizontally. Slopes of the regression lines m with correlation R and significance are specified in Table 1. All regression lines show significant decreasing courses.

Figure 6 shows scatterplots of differences with mean values of the differences of the magnetic and electrical transcranial latencies, EL and ML, with their mean values as a function of the stimulation intensity for the ECR and TC muscle groups. Mean ML and EL and means of paired differences as well as the negative slope, correlation, and significance of the regression lines of the latency reduction as a function of intensity are listed in Table 1. All motor latencies show reductions of the latency times with stimulation intensities for ML between −0.085 and −0.089 ms/% and for EL between −0.09 and −0.107 ms/V. These are highly significantly correlated.

#### Reproducibility of mMEP Motor Latencies

The iso-intensity stimulus-to-stimulus reproducibility and accuracy of ML and EL times, in which case- and intensity-dependent influences are excluded are listed in Table 2. All RP values show higher reproducibility for TES versus TMS. Similarly, all AC values, being linearly related to RP, show for all muscle groups a better accuracy of latency times for TES over TMS.

**TABLE 2:** Survey of the reproducibility, RP, and accuracy, AC, parameters of four muscle groups for TMS and TES.

	**RP_*m*_ ms**	**AC_*m*_ %**	**RP_*e*_ ms**	**AC_*e*_ %**	**RP_*m*_ - RP_*e*_ ms**
ECR L	0.44	2.3	0.31	2.1	0.13
ECR R	0.41	1.9	0.40	1.7	0.01
TC L	0.79	3.0	0.57	1.8	0.22
TC R	1.02	2.3	0.50	1.8	0.52

*RP is the root mean quadratic deviation from of the mean latency value of a latency pair and includes 20 pairs of quadratic terms belonging to four transcranial stimulation intensities at five horses. RP_*m*_ − RP_*e*_ is the mean difference of the reproducibility for comparing TMS versus TES where sig specifies the *p*-value of the significance.*

### Motor Conduction Velocity TES Versus TMS

Table 3 provides an overview of the conduction velocities between stimulation and recording sites for TMS and TES and the intradural fractional length of the enclosed route. The electrical conduction velocities are significantly greater for TES when compared to TMS: ECV_*ECR*_ = 66.17 and ECV_*T*__*C*_ = 73.67 m/s versus MCV_*ECR*_ = 58.47 and MCV_*TC*_ = 63.68 m/s. The ECV values increase to 76.70 and 79.27 m/s after application of the 1.5 ms correction for the MN and 1.0 ms for the neuromuscular delay. A third 1.5 ms delay for a proprioceptive interneuron (PN) yields a further increase to 84.71 and 83.09 m/s.

**TABLE 3:** Survey of the intradural axonal fraction of the total length of the motor conduction route, mean and standard deviations (sd) of the overall TMS motor conduction velocities at TMS (MCV) and TES (ECV), and ECV-MCV differences from MCV from the left side.

**Muscle group**		**Intradural fraction of the motor conduction route**	**MCV**		**ECV-MCV difference**	**ECV**	
			**mean m/s**	**sd m/s**	**mean m/s**	**mean m/s**	**sd m/s**
ECR	inclusive synaptic delays	58%	58.47	2.34	7.70*	66.17	4.68
	exclusive 2.5 ms synaptic delay (MN + NMJ)					76.70	5.44
	exclusive 4.0 ms synaptic delay (PN + MN + NMJ)					84.71	6.01
TC	inclusive synaptic delays	73%	63.68	7.94	9.99*	73.67	4.38
	exclusive 2.5 ms synaptic delay (MN + NMJ)					79.27	4.72
	exclusive 4.0 ms synaptic delay (PN + MN + NMJ)					83.09	4.94

*Significant for *p* < 0.05. To estimate the exclusive total axonal conduction velocities values, EMVs are also given synaptic delay corrections of 2.5 ms (1 ms neuromuscular junction, NMJ), 1.5 ms MN, or 4.0 ms (additional 1.5 ms for PN).

### Comparison of mMEP Amplitudes, TES Versus TMS, and Left Versus Right

No significant difference in mMEP amplitudes between TMS and MEP could be found as depicted in Table 4. Neither was there a significant difference between the left and right sides. Table 4 provides an overview of the mean and standard deviations of mMEP amplitudes within the TCW for both muscle groups and both sides as well as pairwise differences between TMS and TES. There is a high variability of the amplitudes between subsequent measurements for both TMS and TES.

**TABLE 4:** Survey of mean mMEP amplitudes for TMS and TES and their differences between TMS and TES.

**muscle group**		**TMS MAMP**		**TMS-TES difference MAMP-EAMP**			**TES EAMP**		**left-right difference mMEP amplitude**			
		**mean mV**	**sd mV**	**mean mV**	**sd mV**	**sig**	**mean mV**	**Sd mV**	**muscle group**	**mean mV**	**sd mV**	**sig**
ECR	L	3.82	1.32	0.05	1.30	0.93	3.77	132	TMS			
	R	4.83	2.38	0.46	3.34	0.78	4.37	2.49	ECR	−0.61	0.36	0.19
	L and R	4.33	1.26	0.26	3.34	0.85	4.07	2.18	TC	0.26	1.19	0.66
TC	L	4.78	1.29	1.02	1.75	0.26	3.76	1.14	TES			
	R	4.02	1.14	0.52	1.85	0.56	3.50	0.95	ECR	−1.01	2.90	0.48
	L and R	4.40	1.12	0.77	1.58	0.34	3.63	0.86	TC	0.76	0.93	0.14

*The means are computed from mean values per case over all five cases. sd: standard deviation. All differences are not significant for *p* < 0.05.*

## Discussion

Transcranial electrical stimulation and TMS are two different TS techniques used in horses to assess the motor function of the motor tracts in the spinal cord. Both methods are well tolerated under suitable sedation.

Muscle motor evoked potentials from TES and TMS are broadly similar as shown in the four landscapes of Figure 4; nevertheless, there are differences between both techniques that should be kept in mind when interpreting study results. An equivalence between both techniques is the occurrence of extracranially elicited reflexes. These appear specifically in horses as major signal components entangled in transcranial mMEPs. Their characteristics, dominating appearance in waveforms and impact on clinical use of both methods is first discussed, followed by a comparison of mMEP parameters, such as amplitude, MLTs, conduction velocities, and amplitudes for both techniques. This comparison provides a clear view on the physiological background of both techniques in which TES more specifically reflects the motor function of the spinal cord due to a reduced influence of the brain cortex. The latter is shown by a better reproducibility of TES when compared to TMS. A model describing the involved complex neural circuits in TES is included in the discussion.

### Extracranial Elicited Reflexes in TES and TMS

#### Evidence for the Presence of Extracranial Elicited Reflexes Induced by Both TMS and TES in Horses

The current study shows the presence of extracranial elicited reflex activity in both TMS and TES below transcranial motor thresholds. These also exist above transcranial motor thresholds and, thus, are outside the TCW entangled in transcranial mMEPs for both TMS and TES. From a clinical point of view, for TMS, it is especially important to realize that, during the execution of a TMS examination, TMS coil misalignment may go unnoticed because only the extracranial elicited mMEPs are preserved, which, unfortunately, could be misinterpreted as being transcranial mMEPs. These extracranially elicited late mMEPs behave differently when compared to intracranially elicited mMEPs when it comes to an augmented reduction in latency times in answer to increasing stimulation intensities in both TMS and TES.

#### Survey of Possible Involvement of Brain Stem Circuits in the Generation of Extracranial mMEPs

The occurrence of TMS and TES extracranial elicited reflex activity is specific for horses and is described for neither TMS nor TES in primates and human unless under rare pathologic conditions associated with the presence of unsuppressed spinal reflexes and hyperreflexia. In fright animals, however, such as horses, brain stem and spinal reflexes are highly sensitive for sensory input of various origins, and reflex activity may spread out over many segmental levels (Duensing, 1952; Wilkins et al., 1986). In horses, Hahn et al. (1998) reported the bilateral presence of a thoracic cutaneous reflex visible between Th2 (rostral direction) and Th16 (caudal direction) when stimulating at Th6.

The extracranial elicited reflex activity extending over all muscle groups in all extremities and the face appears to be a SR. SRs are known to be generated by involuntary activation of motor tracts located in the brain stem, mainly at the level of the pontomedullary reticular formation, cochlear nucleus, and inferior colliculus. Other reflexes related to the SR are vestibulo-spinal reflexes, laryngeal (LAR), and blink reflexes. In humans and animals, including horses, SRs are usually elicited by auditory and sensory stimuli (Prosser and Hunter, 1936; Duensing, 1952; Davis et al., 1982; Gokin and Karpukhina, 1985; Hori et al., 1986; Wilkins et al., 1986; Brown et al., 1991a,b; Colebatch et al., 1994, 2014; Añor et al., 1996, 1999; Watson and Colebatch, 1998; Álvarez-Blanco et al., 2009; Valls-Sole, 2012; Veres-Nyéki et al., 2012; Sinclair et al., 2017). The reticular system receives multimodal inputs from auditory, sensory, and vestibular origin and also collateral connections from the CST. Reflexes can be modulated mutually by autonomous and heteronymous stimulation of different modalities (Hoffman and Ison, 1980; Ison et al., 1990; Journée et al., 2007; Fitch et al., 2008). SRs evoked by acoustic clicks from TMS coils can interfere with mMEPs in humans (Fisher et al., 2012) and can also be evoked by tactile (Duensing, 1952) and somatosensory electrical stimulation (Gokin and Karpukhina, 1985; Karpukhina et al., 1986; Álvarez-Blanco et al., 2009) though SRs are more difficult to elicit with electrical stimulation in humans and non-fright animals than in horses and may need conditioning. Enhanced sensitivity of SRs to sensory inputs are reported in human pathological conditions, such as the startle disease or hyperreflexia, brain stem reticular reflex myoclonus, and generalized hyperreflexia in postanoxic encephalopathy (Andermann et al., 1980; Brown et al., 1991a; Bakker et al., 2006; Dreissen et al., 2012). SRs are sensitive to modulation by stimuli of various modalities and intensities of which many inhibit and also strongly modulate SR latency (Hoffman and Ison, 1980; Ison et al., 1990; Fitch et al., 2008; Álvarez-Blanco et al., 2009). This agrees with our finding of intensity-dependent decreases in the latency of long latency mMEPs.

In addition, it is known that transcranial stimulation as TMS also activates reticular neurons (Fisher et al., 2012). The high reflex sensitivity in horses makes it theoretically possible that TES and TMS also may be capable of eliciting the SR and other reflexes via intracranial routes. In that case, in horses, transcranial elicited mMEPs possibly may co-carry characteristics of the SR and other reflexes sharing motor pathways to MNs.

#### Influence of TMS and TES Intensities on Late mMEP Latencies

The possibility that middle and late mMEPs are extracranially elicited reflexes from magnetic stimulation in horses has probably been overlooked in the literature. Current explanations for short, middle, and long mMEP latency times are usually focused on differences in conduction velocities of spinal motor tracts (Kawai and Nagao, 1992; Mayhew and Washbourne, 1996; Alstermark et al., 2004, 2007; Nielsen et al., 2007). When reflexes are discussed, these usually address the aftermath of long loop reflexes that follow upon transcranial activation. However, in a previous multipulse TES–mMEP study (Journée et al., 2015), we explained that the late mMEPs evoked by stimuli below the transcranial threshold are most likely ascribed to extracranial excitation of sensory afferents evoking SRs mediated by neural circuits in the brain stem and/or high segmental levels in the spinal cord.

The similarities of the buildup of mMEP wave forms of both fore and hind limb muscle groups agree with our hypothesis that, like TES, TMS also activates extracranially located axons below transcranial motor thresholds and can initiate SR. This may be unique to horses. The extracranial threshold for TMS lies closer to the transcranial threshold than for TES. This may be explained by, (1) for TES, currents induced in the scalp easily spread out through the scalp and pass beyond the ring block with less attenuation so that extracranial sensory axons become activated at relatively low thresholds. This also applies to more remotely located facial motor nerves in which the stimulation threshold of M-responses of the m. caninus in Figure 5A is 14 V, which is about one seventh of the threshold needed for TES to evoke intracranial mMEPs. Late mMEP and M-wave thresholds of the m. CAN are about equal. These are about 8 V lower than the late mMEP thresholds of the ECR and TC. (2) For TMS, the currents induced in the scalp decrease sharply within a few centimeters from the coil (Cohen et al., 1990) so that extracranial activation is constrained to a small rim under the TMS coil just outside the ring block. When no ring block is used, which usually is the case with TMS, then the region where extracranial activation occurs includes the whole surface beneath the coil. This implies that stimulation thresholds for late mMEPs are expected to even be lower than when a ring block is applied. A ring block in TMS may also have a desensitizing influence on extracranially elicited reflexes. The maximum induced current density in the scalp is clearly less with TMS than when TES is used (Cohen and Cuffin, 1991). Due to the relatively low electrical stimulation threshold of late mMEPs, their latency times reach their limit values at the ET level (Journée et al., 2015) while magnetic extracranial thresholds approach their limit values well above MT. This is illustrated in Figure 4. Extra to intracranial (EC-IC) latency differences of the ECR reach a 19-ms limit (Figure 4A), and for TMS, this difference is as high as 34 ms around MT ≈60% (Figure 4B). Similarly, for the TC, the EC-IC latency difference of 38 ms at MT around 35% (Figure 4D) is still 20 ms longer than the 18-ms limit at ET ≈85V (Figure 4C).

The relation between extracranial and intracranial thresholds can be characterized by an extra- intracranial stimulation threshold ratio according to the expectations; the range of the ratio of 67–94% for magnetic stimulation is higher than 22–58% for electrical stimulation. The ring block may partly contribute to the difference.

#### Impact of TMS Coil Misalignment

The occurrence of extracranially elicited late mMEPs is especially important for interpretation of TMS tracings in which coil misalignment can have an additional influence.

Many TMS coils exert a 3-D effect on an area beneath and around the coil to distances of several centimeters (Hess et al., 1987; Rothwell et al., 1987; Cohen et al., 1990; Cohen and Cuffin, 1991; Deng et al., 2013; Fiocchi et al., 2018). Therefore, TMS coils easily get out of transcranial focus so that transcranial MTs rapidly increase and exceed the TMS intensity range. This carries the risk that late mMEPs may be misinterpreted as being from intracranial origin. This is illustrated in a study by Nollet et al. (2003b) in which seven different coil positions were studied in seven horses. The mMEP latencies of two well-placed positions 2 and 7 were in a normal transcranial range, and at misalignments of more than 4 cm at coil positions 1, 3, 4, and 6, the ECR and TC MLTs were increased by more than 15 ms, which is well outside the TCW. This is in accordance with the latency times of extracranially elicited SR.

The electrical field of TES penetrates deeper in the brain at higher stimulation intensities and easily reaches the corticospinal and other spinal bound tracts at the brain stem level although the focus of the electric field becomes increasingly blurred. As a result, the exact location of the TES electrodes becomes less critical at higher stimulation intensities (Burke et al., 1993; Rothwell et al., 1994; Li et al., 2007; Datta et al., 2013; Tomio et al., 2016). Therefore, it is less likely that transcranial mMEPs are missed with TES when compared to TMS.

#### Sensitivity of Extracranial Versus Transcranial Motor Latencies to Increasing Stimulation Intensities

Extracranially elicited mMEPs show a significantly more pronounced reduction in MLT at increasing stimulation intensities when compared to intracranially elicited mMEPs (Figures 4A–D). This is also shown in Figure 2 of Nollet et al. (2003b), where the latencies of late (extracranial) mMEP decrease enormously (by tens of ms) when the TMS intensity only increases from 80 to 100%. This agrees with previous studies (Journée et al., 2015). In Nollet et al., the mean latencies of early (transcranial) mMEPs at well-aligned coil positions 2 and 7 decrease by only 1 ms for the ECR and 2 ms for the TC mMEPs.

Figures 5A–C shows, at gradually increasing TES intensities, a gradual reduction of late mMEP latencies by 15–40 ms of late mMEPs followed by a stepwise transition to early mMEPs with further latency reductions within the 2- to 3-ms range. The latency changes look similar to extracranially elicited SRs from TMS. A possible cause of the suppression or disappearance of late mMEPs during the 20–30 ms after early latency mMEPs is described by de Noordhout et al. (1999), where they argue that it could reflect refractoriness in the MN pools. This suppression is less pronounced in Figures 4A,C.

Nielsen et al. (2007) observed three different groups of mMEP latencies in the biceps brachii and tibial muscles and intracellular recordings of MNs in rats using TMS and selective pyramidal electrical stimulation in the brain stem and demonstrated a dominant role of the reticulospinal tract over the corticospinal tract. At increasing TMS intensities, the mMEPs evolved similarly to our landscape plots in Figure 4B and D by the sub-threshold transcranial appearance of late mMEPs with gradual decreasing latencies over 10 ms.

### Comparison of mMEP Motor Latencies

#### Comparison of Motor Latencies in TMS Versus TES

**FIGURE 7:**
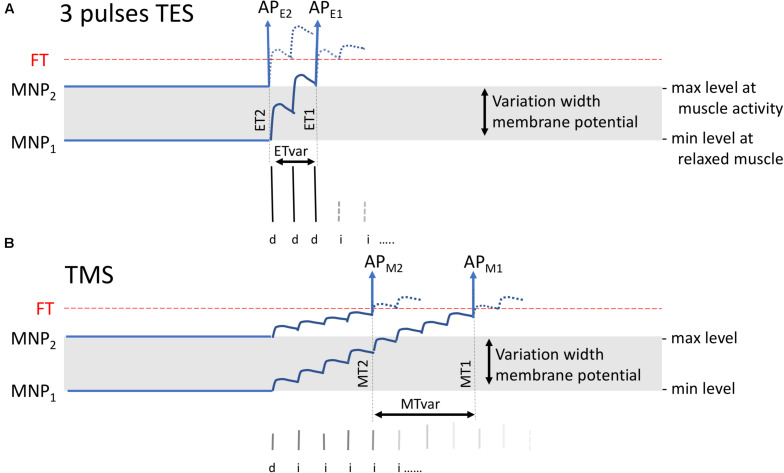
Visualization of expected variations of the MLT for three-pulse TES **(A)** and single-pulse TMS **(B)** when MNPs vary between a resting state at MNP_1_ to a facilitated state at MNP_2_. TES or TMS generate superimposed EPSP stair functions. The model applies to horses when assuming the presence of a corticospinal connection. In practice, the stair-wise course is likely smoothed when synaptic delays of interneurons evolve asynchronously with interpulse intervals. The EPSP stairs result from the contribution of a sequence of D- and I-waves that mainly are transducted by IN and PN but likely less pronounced via monosynaptic connections to MNs as far as these may exist in horses. AP_*E*__1_ and AP_*M*__1_ are the action potentials arising at the FT level that belong to the MNP1 membrane potential while AP_*E*__2_ and AP_*M*__2_ belong to MNP_2_. ETvar and MTvar are the latency variations between ET1 and ET2 and between MT1 and MT2, respectively. The model predicts for TES lower MLTs with smaller variations (ETvar) than in TMS, which is explained from the steep flank resulting from three initial large D-wave amplitudes.

All transcranially elicited mMEP latencies in Table 1 fall within the range of normal MLTs reported in horses in previous studies (Mayhew and Washbourne, 1996; Nollet et al., 2002; Rijckaert et al., 2018). Interestingly, in the current study, MLT values for TMS are about 4 to 4.5 ms higher for the ECR and 5 to 5.5 ms higher for the TC. Remarkable is the wider range of latency times for the TC versus the ECR, which has been reported in previous studies and is confirmed in the current study. This wider range is more pronounced for TMS when compared to TES. Figures 1, 2 in the TES–TMS comparison study in humans of Maertens de Noordhout et al. (1999), show poststimulus time histograms (PSTH), which can be compared with the statistical distribution of latencies in Table 1. The mean PSTH peak duration for the flexor carpi radialis for anodal stimulation is 0.9 ms and is for TMS more dispersed (>5 ms). The ratios of SD values of the four muscle groups of the MLTs in Table 1 for TMS versus TES vary between 1.54 and 1.84 and similarly indicate wider statistical distribution functions. This also agrees with the model in Figure 7 in which the MLT variations, MTvar, of TMS is greater than the MLT variations, ETvar, of TES.

For TMS, the following TC latency times have been reported: 30.2 ± 3.4 ms, *n* = 10 (Mayhew and Washbourne, 1996); 30.5 ± 5.3 ms, *n* = 84 (Nollet et al., 2004); 32.6 ± 2.0 ms, *n* = 6 (Nollet et al., 2003c); 35.9 ± 3.5 ms, *n* = 12 (Nollet et al., 2002); 38.5 ± 3.8 ms, *n* = 10 (Rijckaert et al., 2018), and 39.8 ± 15 ms, *n* = 7 (Nollet et al., 2003b); for TES, the following latency times have been reported: EL_*ECR*_ = 19.4 ± 1.5 ms and EL_*TC*_ = 36.2 ± 2.1 ms (Journée et al., 2018).

Only the approach of the current study, in which both TMS and TES were performed in the same set of horses and on the same occasions, allows for standardized comparison of mMEP parameters between both techniques. Indeed, the reported latency time differences between TMS and TES of a few ms precludes retrospective statistical evaluation of available literature data when, for example, mean ML_*TC*_s can vary over more than 10 ms between horses. The intra-individual TES–TMS comparisons eliminate the influence of factors, such as body size, temperature, or sedation. This is demonstrated in the intra-individual comparisons in Table 1 in which ML - EL differences are highly significant between TMS and TES.

From a physiological comparative point of view, it is interesting to mention that the MLT differences between TMS and TES in horses of, respectively, 4.27 ms for the ECR and 5.31 ms for the TC (Table 1) are greater than a 1- or 2-fold synaptic delay of 1–2.7 ms in human and primates (Hess et al., 1986; Day et al., 1987; Amassian et al., 1989; Nielsen et al., 1995; Ubags et al., 1999; Di Lazzaro et al., 2004). An extra MLT difference between multipulse TES and TMS may result from the difference between the steep flank of the fast-rising EPSP tread stairs resulting from three large D-waves versus the slow rising EPSP stairs of TMS from the low amplitude I-wave series. The slower climbing stairs function predicts an extra time difference of one to several EPSP intervals between ET2 and MT2 or between ET1 and MT1 as depicted in Figures 7A,B.

#### Left–Right Comparison of mMEP Latencies for TMS and TES

When comparing MLTs between left and right for both techniques, no significant differences could be found in any of the studied muscle groups (Table 1). This was also reported for both stimulation techniques in previous studies (Mayhew and Washbourne, 1996; Nollet et al., 2004; Journée et al., 2015; Rijckaert et al., 2018).

#### Dependence of mMEP Latencies on Transcranial Stimulation Intensities of TMS and TES

For both techniques, there is a clear influence of stimulation intensity on MLTs, which is to be expected based upon previous studies (Sylvestre et al., 1993; Nollet et al., 2003a; Journée et al., 2018). mMEP latencies decrease with increasing TES and TMS intensities (Figure 6). According to the slopes of the regression lines, the decay of ML over an intensity increase from 10 to 40% above MT is for the ECR and TC, respectively, −2.6 and −2.7 ms, and the EL latency decays between 10 and 40 V above ET for the ECR and TC are –2.6 and −3.2 ms. For TMS, a bending becomes visible at about MT + 20% and approaches limit values at around MT + 40%. A similar reduction of the ML of the TC is reported in dogs when the TMS intensity is doubled from 50 to 100% (Sylvestre et al., 1993). MT and ET decreases cannot be compared between both techniques in absolute terms because TMS and TES latency times are expressed in different intensity units: respectively, output percentages for TMS and voltages for TES. The MLT regression converges to limit values. The curved course looks similar for TMS in dogs (Sylvestre et al., 1993).

The intensity-dependent latency shifts of 2.6–3.2 ms are explained by the temporal summation of EPSPs from D- and I-waves. At incremental stimulation intensity, D- and I-wave amplitudes increase although additional I-waves may appear as shown in Figure 2A for TMS and in Figure 2C for TES (Edgley et al., 1990, 1997; Burke et al., 1993; Rothwell et al., 1994; Kaneko et al., 1996). Figures 2A–F illustrates that higher EPSP amplitudes of D- and I-waves steepen the flank of the membrane potential so that the FT is reached earlier as shown by the vertical arrows. This implies shortening of the MLTs due to increasing stimulation intensity. The model depicted in Figure 7 also explains the jump-wise shortening of the MLT at increasing TS intensities. This has also been described by Amassian et al. in human and primates, in which mono and bisynaptic connections exist (Amassian et al., 1989). The MLT jumps reflect the time intervals between subsequent D–I and I–I waves. Because mMEPs result from a set of recruited MNs, the decrementing MLT steps are also apparent in the mMEPs in Figures 1C,F as indicated by the arrows. When more synaptic interconnections exist, as expected in horses, then the EPSP stair steps of the expected time function of the MN membrane potential dispersion effects deliver a more a gradual smooth course.

### Comparison of Motor Conduction Velocities in TMS Versus TES

As mentioned previously, TMS and TES have different entry points into the brain. For TMS, cortical neurons are included in the transmission of action potentials toward the subcortical region from which onward shared motor routes start for both TMS and TES. When looking at TMS latency times in Table 1, these are significantly greater when compared to those reported for TES. As previously discussed, these differences can partly be ascribed to additional synaptic delays in the initial part of the TMS motor conduction pathway that includes cortical neurons. This is not the case for TES. Because conduction velocities are calculated as the division of the measured length of the traveled route by the measured latency times, TMS conduction velocities are expected to be lower than TES conduction velocities due to the additional synaptic delay of at least the cortical pyramidal neurons. It is important to realize that calculated conduction velocities actually underestimate net axonal conduction velocities because they include an unknown amount of synaptic delays. Only when the net latency time over a shared axonal segment without synaptic interruptions is exactly known, then the net axonal conduction velocities should be independent of the used TS technique. This is indeed not the case for the compound conduction velocities in Table 3, in which synaptic junctions are included in the traveled motor route. As expected from the higher number of included neural synaptic relay points, Table 3 shows that the conduction velocities of TMS to ECR and also to TC muscles are significantly lower when compared to TES.

Table 3 also shows what the effect is of one or two synaptic delays on the conduction velocities. The conduction velocities are for one single MN and for a combination of an MN plus PN increased by, respectively, 15.9 and 27.2% at the ECR and, respectively, 7.6 and 12.7% at the TC muscle after correction for assumed synaptic delays of 1.5 ms for each neuron and 1.0 ms for a neuromuscular transition. The neuromuscular delay may range between 0.8 and several ms (Reed, 1984; Tiiska and Lagerspitz, 1994; Homan et al., 2018). The difference ECV_*TC*_ - ECV_*ECR*_ of 7.5 ms is, after correction, reduced to 4.6 ms. Using a synaptic delay of 1.5 ms and applying reported spinal cord lengths of this study, net CMCVs become, respectively, 43 ± 10 (for TMS) and 71 ± 9 m/s (for TES). These are lower than those reported in Table 3, and therefore, most probably, not monosynaptic but rather polysynaptic connections are being measured. More research is needed in that respect.

Results of the current study can teach us a lot about axonal diameters in horses. When translating MCVs into axonal diameters, it is important to realize that conduction velocities may be different in sections of the central nervous system (both brain and spinal cord) when compared to sections of the peripheral nervous system. For example, reported peripheral MCVs of the radial nerve in horses (innervates ECR) and median nerve are greater than CMCVs: at distal locations: 84.5 and 76.6 m/s, however, as high as 97.8 and 86.9 m/s at proximal locations (Henry et al., 1979; Henry and Diesem, 1981). When translating these conduction velocities into estimation of axonal diameters, very little is known in horses; only sparse data are available with respect to sensory fibers. For comparison, reported maximum diameters of peripheral sensory nerve fibers are 15 μm (Wheeler and Plummer, 1989; Wheeler, 1990b). The reported mean conduction velocities of palmar sensory nerves in adult horses are, respectively, 68.5 ± 3 m/s (Wheeler, 1990a), 61 m/s (Huntington et al., 1989), and 53.4–67.5 m/s (Zarucco et al., 2010). Keeping in mind that sensory conduction velocities are reported to be about 10–25% lower than published motor velocities, this forecasts maximum diameters of motor axons in equine peripheral nerves of 16–20 μm.

When focusing on intradural and, thus, central conduction velocity, it is emphasized that the main parts of the thoracic and pelvic conduction routes are located within this central part (brain and spinal cord). More specifically 6/10 of the thoracic and 3/4 of the pelvic routes are located intradurally. Intradural axonal conduction velocities are, according to the results in Table 3, expected to range from 60 to more than 100 m/s. According to the Hursh conversion factor (Hursh, 1939), maximum axonal diameters of spinal motor tracts are estimated to be at least 15 to 20 μm or even markedly more in case additional interneurons are involved. To our knowledge, no specific data on axonal diameters of spinal motor tracts in horses are published. Conduction velocities as low as or even lower than 10 m/s are reported in the macaque in axons with a diameter of 1–2 μm ∅. On the other hand, in the same species, a proportion of fast-conducting axons with a diameter of 12–15 μm and corresponding conduction velocity of 80 m/s has been reported (Lemon and Griffiths, 2005). In the goat, which can be viewed as the animal species studied nearest to the horse, the largest diameter fibers measure 5 μm (Verhaart, 1970), which implies conduction velocities of at most 36 m/s. This is three to four times too small for a match with the actual CMCVs reported in the current study and literature (Breazile et al., 1967).

When excluding other than net axonal conduction delays, maximum axonal motor conduction velocities in the spinal cord of horses may easily exceed 100 m/s and forecast the presence of axonal diameters of at least 15 to 20 μm. Breazile and coworkers measured a conduction velocity of 70 m/s between the pyramids and the ventral emerge of motor nerve roots (Breazile et al., 1967). Net axonal conduction velocities are higher when synaptic delays are present.

### Model of the Neural Circuitry That Generates Muscle MEPs From TMS Versus TES in Horses

**FIGURE 8:**
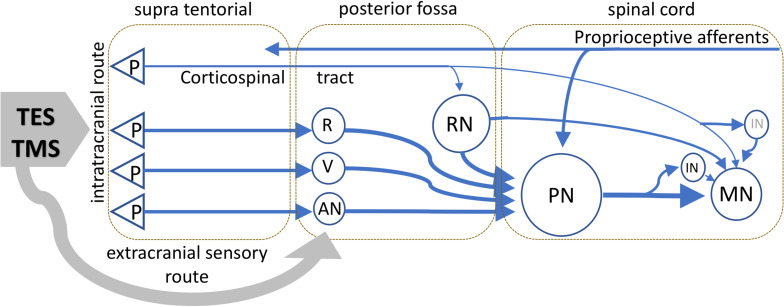
Schematic visualization of the important motor tracts of TS pertaining to TES and TMS as expected in ungulates as horses. The scheme is speculative because no specific anatomic data on horses is available. TMS and TES activate transcranially supra tentorial located axons and also extracranial sensory axons connected to neurons in the brain stem and upper cervical regions. The extracranial connections, as indicated by the gray arrow, are mediated via sensory axons of cranial nerves and possibly high cervical roots and conveyed to the neural network of activated brain stem reflexes. The proprioceptive nucleus PN at C3-C4 has a major integrating role of motor tracts departing from the brain stem from different neurons of which RN are reticular neurons, R the nucleus ruber, V neurons of vestibular nuclei, which receive connections from the pyramidal neurons in the cortex, and AN indicates additional nuclei that mediate other, such as tectospinal, connections from subcortical axons to spinal motor tracts. The PN possibly also receives collaterals from corticospinal tracts. Retrograde connections of the PN to the brain stem and cerebellum are not shown. The MN receive inputs from the PN, RN, and when it applies, directly from the corticospinal or via IN. The possibility of direct connections with MN from remaining brain stem neurons is not incorporated in the diagram. Latency times of TMS and TES reflect the functional tracts that are pivotal in motor control.

The corticospinal tract, which has a dominant role in the voluntary control of movement in primates, becomes less important in phylogenetically older species (Kuypers and Martin, 1982). In many animals, the extrapyramidal spinal motor pathways and associated neuronal circuits are believed to play a major role in the motor control. The absence of monosynaptic connections in both TS techniques implies additional synaptic connections to MNs. This would contribute to even higher net axonal conduction velocities. An outline of main connections from literature data is given in the model of Figure 8. This model is speculative because no specific anatomical data on horses is available.

The model shows the dominating connections from the brain stem to MNs. Although differences may exist between phylogenetically older species such as rodents, cats, rats, and ungulates, such as horses, it is expected that these animals share the integrating and dominating role of the PN in the control of movements. The reticulospinal, rubrospinal, tectospinal, and vestibulospinal tracts are important brain stem–leaving motor tracts and are a prominent input to C2-C4 spinal PNs. The PN is an important common path station. It relays to cervical and thoracic MNs and back to the brain stem and cerebellum (Alstermark et al., 2004, 2007; Nielsen et al., 2007). Collateral connections of the corticospinal tract with reticular and other neurons in the brain stem indirectly provide an access port to the PN. The PN also receives somatosensory axons of which many are of proprioceptive origin. The large variety of connections via the PN and interneurons causes an increasing MN membrane potential in which a monosynaptic transfer of corticospinal D- and I-waves is subordinate or absent. The corticospinal tract is likely not a functionally significant motor pathway in all mammals. For example, in rats, the locomotion is not affected caudally from pyramidal tract lesions (Muir and Whishaw, 1999).

### mMEP Amplitudes, TES Versus TMS, and Left Versus Right

It is important to realize that, in horses, transcranial mMEP waveform and amplitudes can reliably be judged within the TCW, in which contamination by late components is precluded. The remaining large part cannot reliably be analyzed due to the interference with late mMEPs.

No significant differences could be found in mMEP amplitude values between TES and TMS. This is due to the large variability seen between successive amplitude measurements for both techniques. This large variability in measured amplitudes is in great contrast with the small variations seen in latency time measures. Comparable results for TMS amplitudes have been reported by Nollet et al. (2004) and for TES amplitudes by Journée et al. (2018).

The large variability in recorded mMEP amplitudes can be attributed to several factors. Standing horses show often modest varying EMG background activity. This modulates membrane potentials of MNs that also receive additional efferent connections from proprioceptive neurons PN at the level of C3 and C4. PNs are pivotal in the integration of proprioceptive afferent input and connections with reticular neurons in the brain stem and cerebellum. Proprioceptive feedback plays an important role in the control of the standing posture. Figures 7A,B shows the modulating effects of stimulation intensities on latency times for both TMS and TES. Important to notice here is that mMEP amplitudes are also higher at higher stimulation intensities due to increased MN recruitment. Also, other modulating factors may contribute to the large variations of mMEP amplitudes recorded for both TMS and TES in all four muscle groups (Table 3). The trial-to-trial variability of amplitudes varies for TES between 1:1.3 and 1:6.5, and variations between 1:1.4 and 1:13 are observed for TMS. These values are somewhat higher than those reported in anesthetized human patients for stimulation intensities at supramaximal levels (range is 1:1.7 to 1:3.3) and are lower than the large variation of 1:26 at intensities just above ET (Journée et al., 2017). The amplitude variations at supra-maximum intensity agree with those reported in a previous study (Journée et al., 2015).

No left versus right differences could be found for mMEP amplitudes for both techniques for both muscle groups as could be expected based upon previous studies.

### Reproducibility of TMS Versus TES

Unlike the large variability between mMEP amplitudes of subsequent measurements, mMEP latencies show a marked lower variability. This is supported by the standard deviations as depicted in Table 1 in which differences between TMS and TES are evident: sd_*ML,ECR*_ = 1.33 ms > sd_*EL,ECR*_ = 0.84 ms and sd_*ML,TC*_ = 3.48 ms > sd_*EL,TC*_ = 1.89 ms. These values are accompanied by low coefficients of variations, defined as the division of standard deviations by mean values. Coefficients of variation are for multipulse TES smaller than for TMS: CV_*ML,EC*_ = 5.5% > CV_*EL,EC*_ = 4.2% and CV_*ML,TC*_ = 8.2% > CV_*EL,TC*_ = 5.1%. The accuracy “AC” is a variation parameter of subsequent latency pairs belonging to one stimulation intensity. AC is, unlike CVs, designed to be insensitive to case and stimulation intensity and shows similar differences between TMS and TES as CVs: AC_*ML,EC*_ = 1.9–2.3% > AC_*EL,EC*_ = 1.7–2.1% and AC_*ML,TC*_ = 2.3–3.0% > AC_*EL,TC*_ = 1.8% (Table 2). The differences between TES and TMS are also apparent when looking at the reproducibility parameter, RP, which is linearly related to AC: RP_*ML,EC*_ = 0.41–0.44 ms > RP_*EL,EC*_ = 0.31–0.4 ms and RP_*ML,TC*_ = 0.79–1.02 ms > AC_*EL,TC*_ = 1.8%. The TMS versus TES differences show all positive values. These outcomes are in favor of TES over TMS reproducibility showing the highest differences for the left ECR and right TC, a smaller difference for the left TC, and a nearly zero difference for the right ECR.

The reason why the reproducibility of mMEP latencies is better for TES than TMS may be attributed to several different reasons. The most important factor may be the influence of background muscle contraction in TMS. The state of muscle tonus and, thus, muscular contraction obviously has an important effect on the latency time interval between transcranial stimulation and subsequently induced contraction in both stimulation techniques. A higher state of contraction coincides with a higher MN membrane potential as depicted by MNP2 in Figures 7A,B. Due to this, by the extra arousal increased baseline level, during the EPSP summation, the MN membrane potentials reach their FT at an earlier time point. This agrees with literature data reporting a stepwise reduction of latency times during a gradual incrementing course from rest to voluntary contraction. For TMS, the latency of the APB drops by 2.3 ms (Hess et al., 1986). Day et al. (1987) measure magnetic latency reductions of the FDI of more than 3 ms and maximal 1.8 ms for TES using a similar study protocol.

It is expected that motor latencies are modulated by the background EMG in standing horses. The dominating three D-wave train of high amplitudes versus I-wave series of TES with lower amplitudes results in a steeper flank than the more slowly evolving EPSPs induced by TMS. Figure 2D illustrates for a human monosynaptic corticospinal model that the three I-wave amplitudes are 10 to 50% of the initial D-wave amplitude. This means that, for a three-pulse TES train, spontaneous membrane potential variations of MNs may cause latency changes between 1 to maximally 2 EPSP intervals, and the relatively more slowly evolving EPSP stairs of TMS implies a larger variation of more EPSP intervals as depicted in Figures 7A,B. When compared to the MLTs, higher variation widths of MLTs are recognized in the stimulus-to-stimulus reproducibility of electrical latency times RP and accuracy AC.

When summarizing, the reproducibility and accuracy of mMEPs of all four muscle groups is better for multipulse TES when compared to TMS, which is significant, except for one muscle group. The differences may likely be ascribed to a higher sensitivity of MLTs to spontaneous variations of resting motoneuron membrane potentials related to muscle background contractions.

## Conclusion

In horses, TMS and multipulse TES are interchangeable techniques for assessing motor functions of the spinal cord. MLTs are very suitable for that purpose and are, on average, several milliseconds longer (4–5.5 ms) for TMS when compared to TES. TES shows higher reproducibility and accuracy of measures when compared to TMS due to less cascaded interneurons, absence of coil repositioning errors, application of multipulse stimulation, and less sensitivity for variations induced by background muscle tonus. Real axonal conduction times and conduction velocities are most closely approximated by TES. Both TMS and TES induce in horses the occurrence of SRs. These are extracranially induced, occur already at stimulation intensities below the threshold for transcranial stimulation, and can, thus, lead to misinterpretations. To avoid false interpretations, transcranial mMEPs can be identified when they arise at the jump wise shortening of the latency time over 15–20 ms when passing the transcranial motor threshold at increasing stimulation intensities for both TMS and TES. A ring block over the vertex is advised to reduce the interference by extracranial mMEPs.

Transcranial electrical stimulation reflects the functional integrity of neural elements of the transcranial route along brain stem nuclei, extrapyramidal motor tracts, PNs and MNs. The corticospinal tract looks less important for control of movement in equids. This is in great contrast with primates.

## Data Availability Statement

Upon request, the raw data supporting the conclusions of this article will be made available by the authors, without undue reservation.

## Ethics Statement

The animal study was reviewed and approved by animal ethics committee of the University of Groningen, the Netherlands and registered as DEC6440B. Written informed consent for participation was not obtained from the owners because the owners agreed verbal their horse to participate in the study.

## Author Contributions

SJ and HJ contributed equally to this article in study design, application for ethical approval, data acquisition and analysis, interpretation and writing. HB, SR, and CD critical revision important for the intellectual content based on their professional background. CB provision of experimental facilities, giving practical advices and revision of the manuscript. CD involved in concluding stage regarding interpretation, writing and revision. All authors contributed to the article and approved the submitted version.

## Conflict of Interest

The authors declare that the research was conducted in the absence of any commercial or financial relationships that could be construed as a potential conflict of interest.

## Abbreviations

APB: abductor pollicis brevis
CAN: m. caninus
CMCV: central motor conduction velocity
CST: corticospinal tract equivalent of pyramidal tract
D-wave: epidural recorded traveling action potential from direct activation of the CST
ECR: m. extensor carpi radialis
ECV: transcranial electrical motor conduction velocity
EL: transcranial electrical motor latency time
EPSP: excitatory postsynaptic potential
ET: transcranial electrical motor threshold
FDI: first dorsal interosseous
FT: firing threshold
IN: interneurons
I-, wave: epidural recorded traveling action potential from indirect activation of the CST
m: Index muscle group
MCV: transcranial magnetic motor conduction velocity
mDL_*m*_: mean of paired differences ML_*m.n*_ – EL_*m,n*_
MEP: motor evoked potential
ML: transcranial magnetic motor latency time
MLT: motor latency time
mMEP: muscular evoked potential
MN: motoneuron
MNP: motoneuron membrane potential
MT: transcranial magnetic motor threshold
n: index case
PN: propriospinal neuron
SR: startle response
TC: m. tibialis cranialis.
TCW: transcranial time window
TES: transcranial electrical stimulation
TMS: transcranial magnetic stimulation.
